# Adaptive evolution of endothelial nitric oxide synthase (NOS3) may reduce cetacean susceptibility to decompression sickness

**DOI:** 10.1093/molbev/msag144

**Published:** 2026-06-11

**Authors:** Ran Tian, Liang Zhao, Tinghui Li, Yinyin Yan, Inge Seim, Guang Yang

**Affiliations:** Jiangsu Key Laboratory for the Biodiversity Conservation and Sustainable Utilization in the Middle and Lower Reaches of the Yangtze River Basin, College of Life Sciences, Nanjing Normal University, Nanjing, China; Jiangsu Key Laboratory for the Biodiversity Conservation and Sustainable Utilization in the Middle and Lower Reaches of the Yangtze River Basin, College of Life Sciences, Nanjing Normal University, Nanjing, China; Jiangsu Key Laboratory for the Biodiversity Conservation and Sustainable Utilization in the Middle and Lower Reaches of the Yangtze River Basin, College of Life Sciences, Nanjing Normal University, Nanjing, China; Jiangsu Key Laboratory for the Biodiversity Conservation and Sustainable Utilization in the Middle and Lower Reaches of the Yangtze River Basin, College of Life Sciences, Nanjing Normal University, Nanjing, China; Marine Mammal and Marine Bioacoustics Laboratory, Institute of Deep-sea Science and Engineering, Chinese Academy of Sciences, Sanya, China; Jiangsu Key Laboratory for the Biodiversity Conservation and Sustainable Utilization in the Middle and Lower Reaches of the Yangtze River Basin, College of Life Sciences, Nanjing Normal University, Nanjing, China; Southern Marine Science and Engineering Guangdong Laboratory (Guangzhou), Guangzhou, China

**Keywords:** decompression sickness, cetaceans, nitric oxide synthase, eNOS, adaptive evolution

## Abstract

Cetaceans tolerate repeated diving bouts. While the extent to which cetaceans experience decompression sickness remains debated, this group of fully aquatic mammals must have evolved a tolerance to damaging nitrogen (N_2_) gas bubbles that can form during overly rapid ascents or after prolonged dives. Here, we present the first in-depth molecular evolutionary analysis of the nitric oxide synthase gene family across cetaceans and identify cetacean-specific amino acid substitutions in *NOS3* (encoding endothelial nitric oxide synthase) that likely arose in a stem cetacean ancestor, coincident with the transition to obligate aquatic life. Using *in vitro* assays, we demonstrate that cetacean endothelial nitric oxide synthase exhibits enhanced enzymatic activity and function, potentially mediated by strengthened binding to its molecular chaperone Hsp90. Our findings provide a molecular foundation for an evolved, more resilient vascular system in cetaceans, offering new insights into their adaptations to a hyperbaric environment.

## Introduction

Cetaceans (whales, dolphins, and porpoises) are fully aquatic mammals that descend from a semi-aquatic ancestor ∼55 million years ago (Berta et al. 2005; Pyenson 2017; McGowen et al. 2020). As breath-hold divers, cetaceans exhibit specialized diving behavior characterized by bouts of repeated dives interspersed with short surface intervals (Berta et al. 2005). In humans, intravascular and extravascular nitrogen (N_2_) gas bubbles can form on overly rapid ascents or after prolonged dives. This bubble formation, triggered by fluctuations in ambient pressure, causes decompression sickness (DCS, commonly known as “the bends”) (Mahon and Regis 2014). Several mechanisms have been proposed to explain how bubbles exert their damaging effects, including direct mechanical disruption of tissue, occlusion of blood flow, endothelial dysfunction or cell death, ischemia, oxidative stress, and inflammation (Montcalm-Smith et al. 2007; Mitchell 2024; Mrakic-Sposta et al. 2024). Clinically, this manifests as joint pain (“limb bends”), cutaneous eruptions or rashes (“skin bends”), neurological dysfunction, cardiorespiratory symptoms (“chokes”), and in severe cases, shock and death (Francis and Mitchell 2003). Although gas embolism associated with pathological lesions has been documented in stranded cetaceans—notably in beaked whales exposed to mid-frequency naval sonar (Jepson et al. 2003; Fernández et al. 2005)—there is no conclusive evidence that cetaceans naturally experience DCS (Lemaitre et al. 2009; Fahlman et al. 2021; Yuen et al. 2022). An inherent DCS-tolerance in marine mammals is supported by anatomical and physiological studies (see Text S1 for details), but little is known about the underlying molecular, biochemical, and genetic mechanisms.

A palpable evolutionary strategy by cetaceans to mitigate DCS is to employ molecules that directly target the nitrogen gas bubbles that cause it (Fahlman et al. 2021). Among these is nitric oxide (NO), a gas essential for preserving vascular integrity and function (Duplessis and Fothergill 2008). NO and its releasing agents have been shown to reduce intravascular bubble formation and to improve decompression sickness recovery (Mitchell 2024; Schipke et al. 2026). By increasing blood flow, NO may also enhance nitrogen (N_2_) washout from tissues, a process that would promote the shrinkage of gas bubbles (Møllerløkken et al. 2006). As a potent vasodilator, NO increases local blood flow and oxygen delivery. NO also influences local oxygen homeostasis directly by modulating HIF protein stability, thereby supporting cellular survival during hypoxia (Man et al. 2014).

Nitric oxide synthases (NOSs) are enzymes responsible for NO production by catalyzing the oxidation of the amino acid L-arginine with molecular oxygen to form L-citrulline. Three NOS isoforms are highly expressed in specific tissues: neuronal (also known as nNOS; *NOS1*), activated by calcium-dependent calmodulin binding to modulate neurotransmission; inducible (iNOS; *NOS2*), which generates NO during systemic injury via activated leukocytes; and endothelial (endothelial nitric oxide synthase [eNOS]; *NOS3*) in blood vessels (Iova et al. 2023). All NOSs share a bidomain protein structure, in which an N-terminal oxygenase domain containing binding sites for iron protoporphyrin IX (heme), BH_4_, and L-arginine is linked by a calmodulin (CaM)-recognition site to a C-terminal reductase domain that contains binding sites for FAD, FMN, and NADPH (Förstermann and Sessa 2012). The best-known mode of action of NO occurs in the vasculature, where Ca^2+^-dependent activation of eNOS (*NOS3*) in the endothelium produces NO that freely diffuses into vascular smooth muscle cells, binding to heme groups of guanylate cyclase (GC) to enhance cyclic guanosine monophosphate (cGMP) synthesis. Next, cGMP activates protein kinase G (PKG), which phosphorylates downstream targets to inhibit myosin light chain kinase, promoting vasorelaxation and maintaining vascular tone. This signaling cascade underscores the critical role of eNOS in endothelium-dependent vasodilation and cardiovascular homeostasis (Tran et al. 2022).

Here, we hypothesized that changes to one or more cetacean NOS genes underlie improved clearance of harmful gas bubbles. Our computational and experimental findings suggest that enhanced NO production by cetacean eNOS (*NOS3*) reduces intravascular bubble formation and buffers the resulting oxidative stress, revealing a molecular mechanism for the evolution of diving resilience, including protection against decompression sickness.

## Results

### Evolutionary analyses of cetacean NOS genes

**Figure 1:**
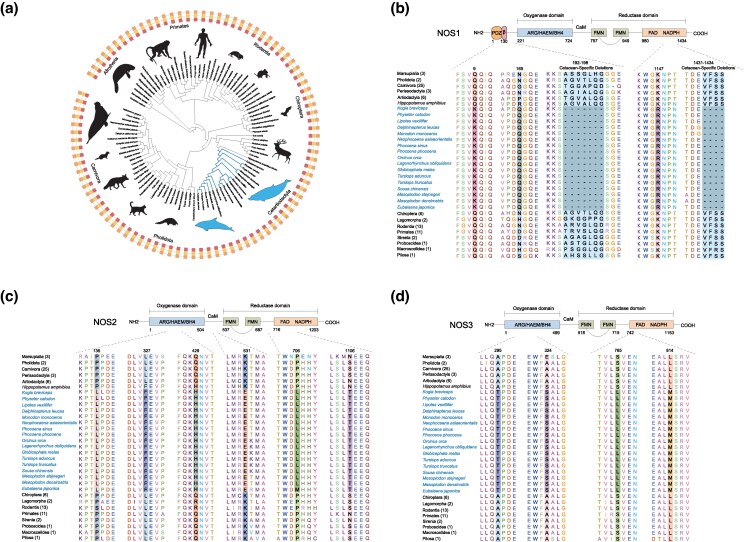
Overview of the dataset and key findings on the evolution of the NOS gene family in cetaceans. (a) Phylogenetic tree of mammalian species (from TimeTree). Colored boxes indicate the presence of *NOS1* (brick-red), *NOS2* (ginger), and *NOS3* (brown) genes. (b–d) Alignment showing amino acid substitution and deletions in cetacean nitric oxide synthase proteins. Cetacean species are indicated in blue font. For noncetacean groups, the numbers of species examined is shown in parentheses. Silhouettes of mammals were taken from phylopic.org.

Coding sequences of *NOS1*, *NOS2*, and *NOS3* were obtained from 94 species, representing major mammalian clades (14 orders) (Table S1). Consistent with (Andreakis et al. 2011) and the essential function of this gene family in mammals, each of the three genes was found to be single-copy in all species examined (Fig. 1a).

Amino acid changes at evolutionarily conserved positions can contribute to speciation and environmental adaptations (Ng and Henikoff 2006). By comparing NOS gene sequences across 17 cetaceans and 77 outgroup species, we identified 13 species-specific substitutions and two in-frame indel events in cetacean NOS genes: *NOS1* harbors three amino acid substitutions (Q9K, P165Q, K1147R) and two amino acid deletion events (in-frame deletion at residues 192–198 and 1431–1434) (Fig. 1b and Figure S1). Six (P135L, L327F, Q429H, K531E, P706L, N1106T) and four (A295T, A324S, S765L, L814M) amino acid substitutions were found in *NOS2* (Fig. 1c and Figure S1) and *NOS3* (Fig. 1d and Figure S1), respectively. We detected no convergent or parallel amino acid substitutions in NOS genes shared between cetaceans and other marine mammals, including fully aquatic sirenians, semi-aquatic pinnipeds, or both.

To detect signatures of selection in the cetacean lineage, we initially employed branch-site and branch models in PAML (Yang 2007). Positive selection was not observed along the cetacean lineage (Table S2); however, accelerated evolution was detected when the cetacean lineage was set as the foreground in the *NOS1* and *NOS2* data set (*NOS1*: *P* = 3.92 × 10^−7^; *NOS2*: *P* = 0.011; Table S3). *NOS3* (*P* = 0.32) showed no evidence of accelerated evolution in PAML. Unlike PAML, HyPhy (Kosakovsky Pond et al. 2020) includes models that account for synonymous rate variation (SRV), uneven d*S* across codons due to factors such as GC content, CpG hypermutability, and recombination. Ignoring SRV can lead to false negatives (and positives) in selection analyses (Davydov et al. 2019; Wisotsky et al. 2020). Applying HyPhy (Kosakovsky Pond et al. 2020), we detected positive selection of all NOS genes using BUSTED and MEME (Tables S4 and S5). The aBSREL model did not detect positive selection of NOS genes at the crown cetacean node (Table S6). However, because aBSREL tests for branch-specific bursts, its power is reduced when adaptive substitutions manifest as weak, pervasive selection across the extant phylogeny or accumulated gradually along lineages not in our dataset (i.e. extinct ancestral branches without genomic data along the semi-aquatic to aquatic transition of cetaceans). The 13 cetacean-specific amino acid substitutions in NOS genes did not overlap the sites under selection in the MEME analysis. Even though these substitutions appear as fixed differences between cetaceans and outgroups in our dataset, they may have become adaptive in extinct ancestors during their semi-aquatic to aquatic transition and selective pressure could have diminished over evolutionary time, making positive selection at these sites undetectable in extant cetaceans (as discussed by (Clark et al. 2025)).

Six cetacean-specific amino acid changes (NOS1: K1147R, del 1431-1434; NOS2: P706L, N1106T; NOS3: S765L, L814M) are in the FAD/NADPH region of the reductase domain responsible for electron transfer to the oxygenase domain. Five (NOS2: P135L, L327F, Q429H; NOS3: A295T, A324S) are located in the ARG/HAEM/BH4 region of the oxygenase domain that binds oxygen and catalyzes stepwise NO synthesis from L-arginine. Three (NOS1: Q9K, P165Q, deletion of 192-198 AA) are in the PDZ domain unique to neuronal NOS1 that targets it to synaptic sites in the brain and skeletal muscle (Förstermann and Sessa 2012). Functional effects of the residue changes were predicted using PolyPhen-2 (Adzhubei et al. 2013) and SIFT (Ng and Henikoff 2003). These tools classified eight (NOS2: P135L, L327F, K531E, P706L, N1106T; NOS3: A295T, S765L, L814M) and four (NOS1: Q9K, P165Q; NOS2: P135L; NOS3: A295T) cetacean amino acid substitutions as intolerant, respectively, suggesting that they result in functional changes (Table S7). Notably, two cetacean substitutions (NOS2: P135L; NOS3: A295T) located in the oxygenase domain were predicted to affect protein function by both tools.

### Functional assessment of cetacean *NOS3*

**Figure 2:**
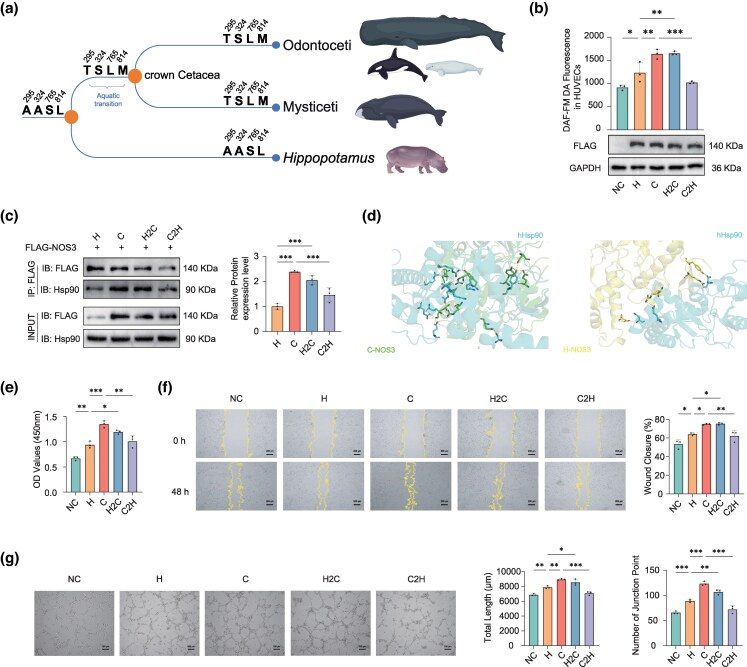
Functional and interactome comparison of cetacean and human NOS3. (a) Phylogenetic tree illustrating the evolution of four cetacean-specific amino acid substitutions in NOS3. The terminal taxa shown are the extant fully aquatic suborders Odontoceti (toothed whales) and Mysticeti (baleen whales), and the semi-aquatic hippopotamus. Terminal nodes are indicated by blue circles, internal nodes in orange. The blue bracket indicates that the NOS3 substitutions arose somewhere along the lineage leading to fully aquatic crown cetaceans. Extant cetacean and hippopotamus illustrations by Zhenyakot/Shutterstock.com and Hennadii + H/Shutterstock.com, respectively. (b) Top: Nitric oxide synthase activity in HUVECs, measured by DAF-FM DA dye fluorescence. Data are presented as mean arbitrary density units ± SD of *n* = 3 independent experiments, normalized to the human NOS3 (H) construct, with individual data points overlaid. Statistical significance was determined by one-way ANOVA with Tukey's multiple comparisons test (****P < 0.0001, ***P < 0.001, **P < 0.01, *P < 0.05; full details in Table S8). Bottom: Corresponding immunoblot showing FLAG-tagged NOS3 constructs and GAPDH loading control, confirming equal NOS3 expression. Molecular weight markers (kDa) are indicated. Here and elsewhere, NC denotes empty vector control (*pcDNA3.1*); H denotes human *NOS3*, C, cetacean *NOS3*; the expression constructs H2C and C2H code for NOS3 proteins where the four cetacean-specific amino acids in Fig. 1d were replaced by their corresponding counterparts. Full images of blots in Figure S7. (c) Co-IP of FLAG-tagged NOS3 constructs with endogenous HSP90 in HUVECs. Presentation as in (b). Full images of blots in Figure S7. (d) Structural models of human HSP90 (hHSP90AA1) in complex with cetacean (C-NOS3, left) or human (H-NOS3, right) NOS3, predicted by HDOCK and visualized using PyMOL. Yellow dashed lines indicate predicted hydrogen bonds. (e) Cell proliferation of transfected HUVECs measured by CCK-8 assay. Data are presented as mean ± SD from three independent experiments. (f) Scratch wound healing assay of transfected HUVECs. Left, representative images at 0 and 48 h postscratch. Right, quantitative analysis of wound closure (mean ± SD). (g) Tube formation assay of transfected HUVECs. Left, representative images of capillary-like networks taken 8 h postseeding. Right, quantification of total tube length and number of junction points (mean ± SD). e–g, Statistical analysis was performed as in (a), and data points represent biological replicates. The detailed raw data are provided in Table S9.

We decided to investigate the function of cetacean endothelial NO (*NOS3*). The gene has a well-established, nonredundant role in regulating vascular tone and blood pressure (Förstermann and Sessa 2012; Tran et al. 2022). It is regulated by factors such as hypoxia (Østergaard et al. 2007; Pooja et al. 2018, 2020) and vascular shear stress (Chatterjee and Catravas 2008). To assess the cetacean-specific amino acid substitutions (Fig. 1d)—which we estimated to have emerged in the fully aquatic immediate prehistoric ancestors of crown cetaceans (Fig. 2a)—we utilized human umbilical vein endothelial cells (HUVECs), a model for NOS3 activity and regulation due to their endogenous expression of the gene and physiological relevance to endothelial function (Bosma et al. 2023). We constructed expression plasmids for four *NOS3* variants: human (H-NOS3), cetacean (bottlenose dolphin; C-NOS3), and reciprocal chimeras in which the four cetacean-specific residues were swapped to generate a “humanized” cetacean sequence (C2H-NOS3), and a “cetaceanized” human sequence (H2C-NOS3). Western blotting showed similar NOS3 protein expression across the different groups (Fig. 2b). Consistent with the functional impact of cetacean-specific residues, both the native cetacean *NOS3* (C-NOS3) and the cetaceanized human variant produced significantly more NO than their counterparts (C-NOS3 vs. H-NOS3: *P* < 0.05; cetaceanized human vs. native human: *P* < 0.01; Tables S8 and S9). In contrast, humanized cetacean *NOS3* had a significantly lower NO production compared to cetacean *NOS3* (C2H-NOS3 vs. C-NOS3: *P* < 0.01; Table S6). These results suggest that the cetacean-specific amino acid substitutions enhance the catalytic activity of NOS3.

Next, we investigated the molecular basis for the enhanced cetacean NOS3 activity. NOS3-derived NO production is tightly regulated by interactions with three key proteins: the inhibitory scaffold caveolin-1 (i.e. it is a negative regulator of NOS3), the calcium-sensitive activator calmodulin (CaM), and the chaperone heat shock protein 90 (Hsp90) (Fleming and Busse 1999). Under basal conditions, caveolin-1 binds the oxygenase domain of NOS3, maintaining the enzyme in an inactive state. Upon cellular stimulation, increased intracellular Ca^2+^ promotes CaM binding and displaces caveolin-1 to induce a conformational shift that activates electron transfer from the NOS3 reductase domain. Hsp90 stabilizes this active conformation, enhances CaM binding, and facilitates efficient NO synthesis. We hypothesized that the cetacean-specific substitutions in NOS3 might alter its affinity for these critical regulators, particularly its release from caveolin-1 inhibition or its stabilization by Hsp90. We performed co-immunoprecipitation (Co-IP) assays in two cell lines overexpressing cetacean or human *NOS3* forms: CaM was immunoprecipitated from COS7 cells, a fibroblast cell line that expresses high levels of the protein (Wang et al. 2023); Hsp90 from HUVECs. There was no difference in CaM-binding between cetacean and human NOS3 forms (Figure S2). This result is somewhat expected, as no cetacean-specific amino acid substitutions were identified in the CaM-binding region (amino acids 493–512). Cetacean NOS3 displayed increased affinity for Hsp90 compared with the human ortholog (Fig. 2c). Reciprocal site-directed mutagenesis strengthen that this enhanced interaction was directly mediated by the four identified cetacean-specific amino acid substitutions. However, the interaction between NOS3 and Hsp90 is complex and difficult to resolve from sequence data alone (Balligand 2002). We therefore predicted protein–protein interactions using the computer tool HDOCK (Yan et al. 2017), with RossettaDock (Lyskov and Gray 2008; Yan et al. 2017) to optimize the local geometry. The proposed models indicate that cetacean NOS3 and cetaceanized human NOS3 have a stronger hydrogen bonding interaction with Hsp90 (Fig. 2d and Figure S3). There was no significant difference in the predicted binding energy between native and mutant NOS3 forms and CaM (Figure S4).

Gas bubbles formed during dives impairs endothelial integrity and function (Brubakk and Møllerløkken 2009). HUVECs overexpressing the cetacean and cetaceanized human *NOS3* showed increased proliferation and migration (Fig. 2e, f). For a more direct measure of NOS3 function, we performed a tube formation assay, which quantifies the downstream morphogenesis of endothelial cells into capillary-like networks (Arnaoutova et al. 2009). Quantitative analysis of capillary-like tubular structures showed that the total length and number of junction points of the cetacean and cetaceanized human *NOS3* was significantly increased compared with the human and humanized cetacean forms (Fig. 2g).

**Figure 3:**
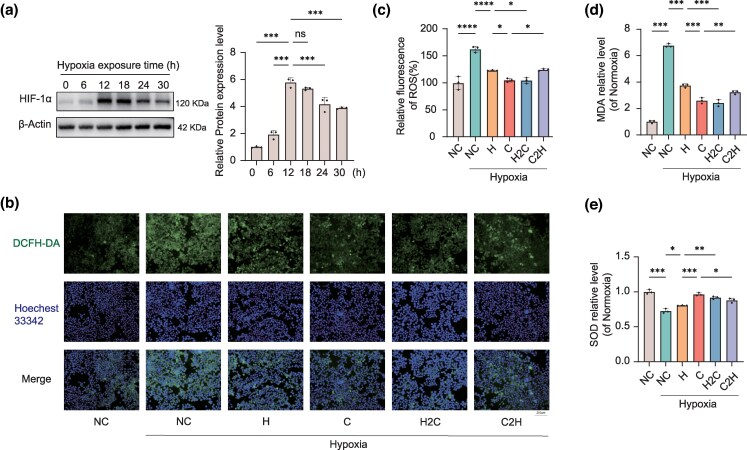
Cetacean NOS3 attenuates hypoxia-induced oxidative stress. (a) Confirmation of HIF-1α stabilization under chronic hypoxia. Left, immunoblot of HIF-1α (HIF1A) in HUVECs exposed to hypoxia (1% O_2_) for the 0 to 30 h. Protein levels were normalized to β-actin (ACTB). Right, densitometric quantification from three independent experiments (mean ± SD). Statistical significance was determined by one-way ANOVA with Tukey's multiple comparisons test (****P < 0.0001, ***P < 0.001, **P < 0.01, *P < 0.05; full details in Table S9). Full images of blots in Figure S7. (b) Fluorescence images of the ROS indicator DCFH-DA in HUVECs cultured under normoxic (21% O_2_) or hypoxic (1% O_2_) conditions for 12 h. Scale bar, 200 µm. Here and elsewhere, NC denotes empty vector control (*pcDNA3.1*), H, human *NOS3*; C, cetacean *NOS3*; the expression constructs H2C and C2H code for NOS3 proteins where the four cetacean-specific amino acids in Fig. 1d were replaced by their corresponding counterparts. (c–e) Quantitative analysis of oxidative stress markers corresponding to the experimental groups shown in (b): (c) DCFH-DA fluorescence intensity. (d) Malondialdehyde (MDA) levels. (e) Superoxide dismutase (SOD) activity. Data are presented as mean ± SD from three independent biological replicates (*n* = 3). Statistical analysis performed as in (a). The detailed raw data are provided in Table S9.

A diving-related gas embolism occurs when gas bubbles enter the bloodstream or tissues, blocking blood flow and causing ischemia (oxygen deprivation). This initial injury can then trigger damaging secondary effects (Mitchell et al. 2022). To mimic the effect of bubble-induced ischemia, HUVECs were subjected to hypoxic conditions (i.e. 1% O_2_) in an anoxic incubator. The protein expression of the hypoxia marker HIF-1α increased gradually, peaking at 12 hours of hypoxia exposure (Fig. 3a). Hypoxic stress disrupts cellular homeostasis, exacerbating damage and leading to the accumulation of intracellular reactive oxygen species (ROS). As expected, hypoxia increased ROS levels compared to the normoxic control (Fig. 3b, c). This increase was attenuated by *NOS3* overexpression, an effect that was more pronounced with the cetacean and cetaceanized human constructs. Consistent with this oxidative stress profile, the two constructs exhibited significantly lower malondialdehyde and higher superoxide dismutase levels (Fig. 3d, e).

**Figure 4:**
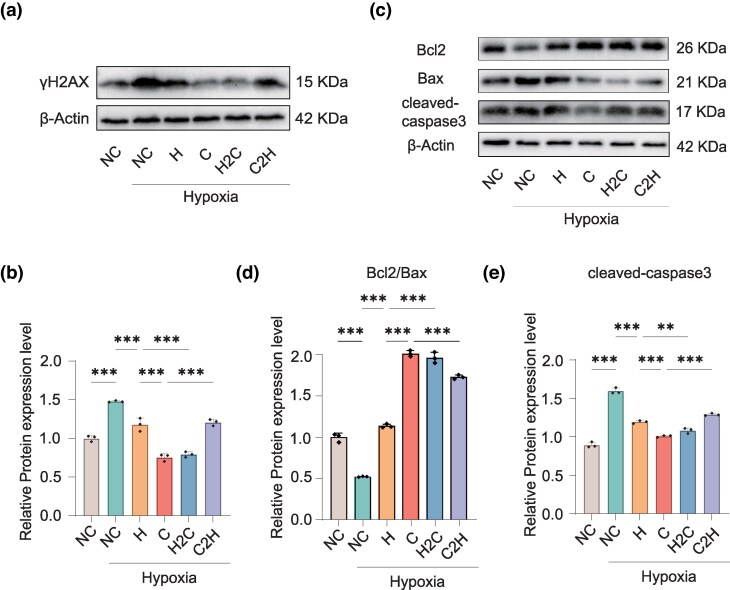
Cetacean NOS3 show reduced hypoxia-induced DNA damage and apoptosis. a) Immunoblot of γH2AX (γ-phosphorylated H2AX) in HUVECs exposed to hypoxia (1% O_2_) for 12 h. Protein levels were normalized to β-actin (ACTB). Here and elsewhere, NC denotes empty vector control (*pcDNA3.1*), H, human *NOS3*; C, cetacean *NOS3*; the expression constructs H2C and C2H code for NOS3 proteins where the four cetacean-specific amino acids in Fig. 1d were replaced by their corresponding counterparts. Full images of blots in Figure S7. b) Densitometric quantification from three independent experiments (mean ± SD) corresponding to (a). Data presented as mean arbitrary density units ± SD, normalized to empty vector control at normoxia (i.e. 21% O_2_), with individual data points overlaid. Statistical significance was determined by one-way ANOVA with Tukey's multiple comparisons test (****P < 0.0001, ***P < 0.001, **P < 0.01, *P < 0.05; full details in Table S8). Protein levels were normalized to β-actin. c) Immunoblot showing key apoptosis-related markers in HUVECs after 12 h of hypoxia (1% O_2_): the antiapoptotic Bcl-2, the proapoptotic Bax, and the apoptosis effector caspase 3. Full images of blots in Figure S7. (d, e) Densitometric quantification of the immunoblot in (c), performed as in (b). The Bcl-2/Bax ratio is a measure of cellular fate, with a higher ratio indicating inhibition of apoptosis. The detailed raw data are provided in Table S9.

ROS promotes DNA damage and apoptosis, ultimately compromising vascular integrity. In endothelial cells, hypoxia-induced oxidative stress is a primary driver of DNA double-strand breaks (DSBs)—particularly harmful lesions that can lead to genomic instability, senescence, and apoptosis (Economopoulou et al. 2009). We assessed the impact of NOS3 on DSBs repair by γ-phosphorylated H2AX (γH2AX) immunoblotting. HUVECs overexpressing *NOS3* had lower γH2AX expression under hypoxia, indicative of reduced DNA damage (Fig. 4a, b). Cetacean and cetaceanized human *NOS3* showed a shared and significant reduction in γH2AX compared to the other constructs. Consistent with increased expression of NOS3 inhibiting apoptosis, we observed a shift in apoptosis-related markers in HUVECs under hypoxia—specifically, an increased Bcl-2/Bax ratio and a reduced caspase 3 level (Fig. 4c–e and Figure S5). This antiapoptotic pattern was again more pronounced with the cetacean and cetaceanized human *NOS3* constructs.

## Discussion

While the extent to which cetaceans experience DCS remains debated (see discussion in the Introduction), this group of fully aquatic mammals must have evolved some tolerance to N_2_ gas bubbles. To contribute to this discourse, we here performed the first in-depth study on the molecular evolution of NOS genes in cetaceans, combining sequence analyses and cell-based functional assays.

**Figure 5:**
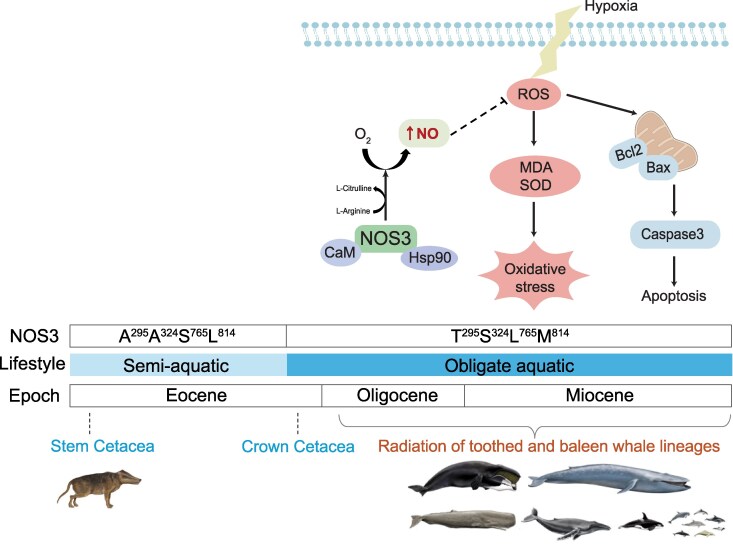
Cetacean-specific amino acid substitutions in endothelial NOS (NOS3) may confer a resilient vascular system. Top, cetacean NOS3 shows enhanced Hsp90 binding and increased NO production in in endothelial cells, reducing (indicated by a dotted closed line) hypoxia-induced oxidative stress, DNA damage, and apoptosis. Bottom, location of four cetacean-specific amino acid substitutions in extant cetaceans, with inferred residues in obligate aquatic stem cetaceans (mid-to-late Eocene) and earlier, transitional stem cetaceans (e.g. *Pakicetus*). Illustrations of *Pakicetus*, and extant cetaceans courtesy of Encyclopædia Britannica, Inc.

We used the classic HUVECs model to assess the function of cetacean *NOS3*. Our results on cetacean, human, and reciprocal site-directed mutants suggest that cetacean NOS3 produces more nitric oxide, potentially by improved binding to its activating protein, Hsp90. Complementary findings include increased cellular proliferation, migration, and tube formation, coupled with decreased DNA damage and apoptosis. Collectively, these findings point to a critical role for NOS3 in conferring cetacean tolerance to decompression sickness (Fig. 5).

Studies on cetaceans to date indicate that certain systems—such as the integument (including hair loss)—are predominantly characterized by gene loss events rather than unique amino acid substitutions; although some cetacean-specific and convergent marine mammal substitutions have been reported (e.g. Foote et al. 2015; Xu et al. 2025; and see appraisal in study by Clark et al. [2025]). The three-gene NOS system is evolutionarily ancient, originating in early vertebrates approximately 450 to 500 Mya (Andreakis et al. 2011). Given that NOS enzymes function as essential catalytic components of physiological pathways rather than as structural elements (e.g. keratins) or transcription factors, we speculate that adaptive evolution in NOS genes would most likely involve function-altering amino acid substitutions rather than gene loss or regulatory changes. We here identify four amino acid changes (A295T, A324S, S765L, and L814M) in cetacean eNOS encoded by *NOS3*. Genetic variants contribute to human population differences in NOS3 function (Ahsan et al. 2006; Wang et al. 2010). A missense mutation (E298D) in exon 7 is associated with decreased nitric oxide levels and is underrepresented in high-altitude-adapted populations (van's Gravesande et al. 2003; Ahsan et al. 2005; Droma et al. 2006; Wang et al. 2009). The cetacean A295T substitution is close to this mutation (Figure S6). Site-directed mutagenesis studies have also identified residues important to NOS function. The cetacean-specific A324S substitution flanks residue 321, deemed critical for maintaining the Hsp90–eNOS interaction (Xu et al. 2007). Hydrophobic residues in residues 326–333 are also essential for β-actin binding, an interaction that shifts NOS3 toward increased NO production (reviewed by Su [2014]).

Increasing the level of nitric oxide is a recurring strategy in nature. Elevated nitric oxide driven by changes in NO synthesis-related genes have been attributed to high-altitude hypoxia tolerance in Tibetan chickens (Peng et al. 2012), humans (He et al. 2018), and fishes (Wang et al. 2020). *NOS3* is under positive selection in the hypoxia-tolerant high-altitude-adapted Tibetan antelope (*Pantholops hodgsonii*) and American pika (*Ochotona princeps*) (Ge et al. 2013), species that emerged within the last 2 to 5 million years. NOS3 regulates blood flow, vascular tone, and oxygen delivery. Both high-altitude hypoxia (hypobaric conditions) and deep-diving ischemia-reperfusion (hyperbaric conditions) challenge the vascular system. Thus, a NOS3-mediated adaptation in cetaceans may reflect the vascular demands of diving (e.g. managing blood flow during breath-hold, preventing ischemia-reperfusion injury), which is functionally analogous to hypoxia adaptation in high-altitude taxa but physiologically distinct from it. In the context of diving, managing blood flow helps reduce nitrogen bubble formation and mitigate hypoxia. The absence of a strong *NOS3* selection signal acting on the branch leading to crown Cetacea (see Results) may reflect an earlier, more intense episode of selection in a cetacean ancestor that optimized the gene for function, subsequently placing it under strong ongoing purifying selection. We hypothesize that the four NOS3 amino acid residues unique to cetaceans were present in an ancestor of crown cetaceans and by parsimony, likely also characterized the physiology of the obligate aquatic late Eocene *Basilosaurus* ∼43 Mya (Pyenson 2017).

In summary, our study reveals that cetaceans have evolved a more efficient endothelial nitric oxide synthase gene, positioning it as key to their physiological adaptations for diving.

## Materials and methods

### Sequence acquisition and alignment

We obtained coding sequences (CDS) of nitric oxide synthase genes (*NOS1*, *NOS2*, and *NOS3*) of 94 mammalian species from NCBI (https://www.ncbi.nlm.nih.gov) and OrthoMaM v12a (Allio et al. 2024). The CDS of human genes were also used as BLAST queries against the genomes of 17 cetacean species (covering both toothed whales [Odontoceti] and baleen whales [Mysticeti]) and other mammalian species to determine whether these genes are single-copy or multi-copy. All NOS sequences analyzed in this study are listed in Table S1. Sequences for each gene in the final data set were aligned using MUSCLE (Edgar 2004) implemented in MEGA7 (Kumar et al. 2016).

### Molecular evolution analysis

Selection pressures on cetacean NOS genes were assessed using codon-based models in PAML v4.7 (Yang 2007). Species tree from TimeTree was used as input (Kumar et al. 2017). We used branch-site and branch models in PAML to test for episodic positive and accelerated selection along the cetacean lineage. We also analyzed positive selection with MEME, BUSTED (Murrell et al. 2012), and aBSREL (Smith et al. 2015) models of HyPhy v2.5.58 (Kosakovsky Pond et al. 2020) for orthologs. BUSTED uses a model incorporating SRV for detecting signatures of episodic diversifying selection in genes, which allows branch-to-branch variation across the entire tree or foreground lineages, and it uses a likelihood ratio test to compare a model including selection (ω > 1 at a proportion of sites) with one that does not (Murrell et al. 2012; Wisotsky et al. 2020). MEME (Mixed Effects Model of Evolution) employs a mixed-effects maximum likelihood approach to test the hypothesis that individual sites have been subject to episodic positive or diversifying selection (Murrell et al. 2012). aBSREL (adaptive branch-site random effects likelihood) inferring the optimal number of ω classes for each branch was used to detect episodic diversifying selection on specific branches in a phylogenetic tree (Smith et al. 2015).

We next screened for cetacean amino acid (AA) site changes using FasParser v2.13.0 (Sun 2017). Identified amino acid residues were classified based on charge, polarity, and volume according to Zhang (2000); assigned into protein domains using the Pfam database v37.2 (Coggill et al. 2008); and their effect on protein function predicted using PolyPhen-2 (Adzhubei et al. 2013) and SIFT (Ng and Henikoff 2003) with the human sequence as the query. The ancestral proteins of NOS genes were reconstructed using PAML codeml; for details, see (Isogai et al. 2018; Chisuga et al. 2025). The empirical model with the JTT matrix was used for the substitution model. No site partition was defined, and the substitution rate was uniform over the sites.

### Structural prediction

To assess the impact of cetacean-specific amino substitutions, three-dimensional structural models were generated using AlphaFold3 (Abramson et al. 2024). Protein–protein interactions between NOS3 and Hsp90 (human isoform HSP90AA1) or CaM (human isoform CALM1) were examined using HDOCK (Yan et al. 2017), with RossettaDock v4.0 (Lyskov and Gray 2008; Yan et al. 2017) to optimize local geometry. Structural visualization was performed using PyMOL v3.1 (DeLano 2002).

### Cell culture

HUVECs were cultured in Roswell Park Memorial Institute medium (RPMI; Senbeijia Biological Technology) with 10% FBS. The African green monkey kidney cell line COS7 was maintained in Dulbecco's modified Eagle's medium (DMEM; Senbeijia Biological Technology, Nanjing, China) supplemented with 10% fetal bovine serum (FBS, Senbeijia Biological Technology), 1% penicillin-streptomycin-amphotericin B solution (100×, Senbeijia Biological Technology).

### Overexpression of *NOS3* in HUVECs and COS7 cells

FLAG-tagged *NOS3* (eNOS) expression constructs were generated by individually subcloning four DNA sequences synthesized by General Biol (Anhui, China) into the *pcDNA3.1* vector (Invitrogen): the protein-coding region of a cetacean (the bottlenose dolphin, *Tursiops truncatus*; construct C-NOS3) and human (construct H-NOS3), as well as cetacean (T295A, S324A, L765S, and M814L; C2H-NOS3) and human (A295T, A324S, S765L, and L814M; H2C-NOS3) sequences where four amino acid substitutions were changed to their reciprocal residues. An empty *pcDNA3.1* vector was used as a control. Transfection was carried out using Lipofectamine 2000 (Thermo Fisher Scientific). Briefly, cells were seeded in 6-well plates and cultured overnight to reach 70% to 80% confluency. Plasmid DNA and transfection reagent were separately diluted in serum-free medium, mixed and incubated for 10 min at room temperature. The DNA–lipid complexes were then added dropwise to the cells, which were further cultured for 24 to 48 hours. Overexpression was confirmed by immunoblotting using mouse monoclonal anti-FLAG (Proteintech cat. no #66008-4-lg at 1:25,000 dilution), with GAPDH (Proteintech #60004-1-lg at 1:200,000 dilution) as a loading control. After three washes with TBST, membranes were incubated with HRP-conjugated secondary antibodies and visualized using enhanced chemiluminescence (ECL) reagents (Vazyme, Nanjing, China) on a ChemiDoc MP Imaging System (Bio-Rad). Quantification of protein expression levels was performed by measuring the gray value intensity of protein bands, normalized to the loading control, using ImageJ software. Results shown are representative of three independent experiments.

### Assessment of *in vitro* nitric oxide generation

Intracellular nitric oxide release was measured using a NO fluorescence probe (cat. no S0019, DAF-FM DA, Beyotime Biotechnology). Briefly, HUVECs were inoculated into 24-well plates in DMEM. After 48 hours of culture posttransfection with *pcDNA3.1* constructs, the medium was discarded, and cells were incubated with 10 μM DAF-FM for 20 min at 37 °C, followed by three PBS washes. The fluorescence intensity was measured by a microplate reader (Biotek Synergy H1, USA) at excitation/emission wavelengths of 488/525 nm.

### Co-immunoprecipitation (Co-IP) assay and western blot analysis

Two days after transfection, HUVEC and COS7 cells were lysed in Western IP Cell Lysis buffer (Beyotime, Shanghai, China) supplemented with 1 mM PMSF (Biosharp) and subjected to co-immunoprecipitation. In total, 10% of the cell extracts were retained for input. Cell lysates were incubated with anti-FLAG magnetic beads (HUABIO, Hangzhou, China) at 4 °C overnight. After washing three times, the precipitates were resuspended in SDS–PAGE sample buffer, boiled for 5 min, and run on a 6% SDS–PAGE gel. The separated protein bands were then transferred onto a polyvinylidene fluoride (PVDF) membrane by electrophoresis. Membranes were blocked with 5% nonfat milk powder in TBST buffer at room temperature for two hours and probed with primary antibodies against FLAG (Proteintech #66008-4-lg at 1:25,000 dilution) for both cell lines, calmodulin (Proteintech #28270-1-AP at 1:700 dilution) for COS7 cells, and Hsp90 (Proteintech #13171-1-AP at 1:7,000 dilution) for HUVEC cells, and ACTB (β-actin; Proteintech #60004-1-lg at 1:200,000 dilution) as a loading control. Membranes were washed, incubated with secondary antibody, and visualized as outlined above.

### Cell proliferation assay

After transfection of HUVECs for 24 hours, cell proliferation was evaluated using a Cell Counting Kit-8 (CCK8, Vazyme, Nanjing, China), a colorimetric assay that quantifies viable cells based on their ability to reduce the tetrazolium salt WST-8 to a formazan dye. Briefly, 100 μL of CCK8 reagent was added to the cell culture medium, and cells were incubated at 37 °C, 5% CO_2_ for 1 hour to facilitate formazan production. Absorbance at 450 nm was measured using the microplate reader (Biotek Synergy H1, USA).

### Cell migration assay

One day after transfection, a consistent straight scratch was made across the HUVECs layer using a p200 pipette tip. The cells were gently washed with PBS to remove cell debris and nonadherent cells generated during the scratch process and replenished with RPMI medium. Images of the scratch area were captured immediately (0 h) and after 48 h using an inverted microscope. Cell migration was quantified by importing images into ImageJ. The migratory capacity of cells was calculated as [(Initial scratch area-Final scratch area)/Initial scratch area] × 100%.

### Tube formation assay

A Plurigel Matrix (Vazyme, Nanjing, China) was thawed at 4 °C. Using a precooled pipette, 20 μL of the thawed matrix was added to each well of a 24-well plate, and incubated at 37 °C for 1 h to polymerize, forming a 3D gel network. HUVECs, pretransfected with plasmids, were seeded into a 24-well plate at 1 × 10^5^ cells/well. The plate was incubated in a cell culture incubator at 37 °C and 5% CO_2_ for 8 hours, during which endothelial cells adhered to the matrix and organized into capillary-like vascular networks. Images were captured in five random fields using a ZEISS Vert.A1 camera mounted on a light inverted microscope. Quantitative analysis of tube formation was performed in ImageJ by counting junction points as a proxy for angiogenic potential.

### Comparative analysis of cetacean and human NOS3 under hypoxic conditions

HUVECs were seeded in six-well plates and transfected individually with H-NOS3, C-NOS3, H2C-NOS3, and C2H-NOS3. Thirty-six hours after transfection, the cells were subjected to hypoxic conditions in a Maworde GC-CT cell chamber (1% O_2_, 94% N_2_, 5% CO_2_) for 12 to 24 hours. Cells were then washed with PBS and incubated with 10 μM DCFH-DA in PBS for 30 min at 37 °C. To visualize nuclei, the cells were subsequently washed with PBS and stained with Hoechst 33342 solution (cat. no C1026, Beyotime, Nanjing) for 40 min at 37 °C. Fluorescent images were captured at 488 nm (excitation) and 525 nm (emission) wavelengths using a fluorescence microscope (Axio Imager M2). Image intensity was quantified using ImageJ and statistically analyzed using GraphPad.

Commercial kits were employed to assess intracellular reactive oxygen species (ROS; cat. no S0034S, Beyotime, Nanjing), malondialdehyde (MDA; A003-4-1, Jiancheng Bioengineering Institute, Nanjing, China), and superoxide dismutase (SOD; A001-3-2, Jiancheng Bioengineering Institute, Nanjing, China). Following 12 h of hypoxia, immunoblotting was performed to assess key markers of DNA damage and apoptosis. The DNA repair marker γ-phosphorylated H2AX (γH2AX; Cell Signaling Technology #2577S at 1:1,000 dilution) and apoptosis-related proteins Bax (HUABIO, ER0907 at 1:10,000 dilution), Bcl-2 (HUABIO ET1702-53 at 1:10,000 dilution), and cleaved caspase 3 (Proteintech 82707-13-RR at 1:25,000 dilution) were analyzed according to the standard immunoblot protocol detailed earlier. The gene expression of Bax (*BAX*), Bcl-2 (*BCL2*), and caspase 3 (*CASP3*) were also evaluated by quantitative reverse transcription PCR across hypoxia time points (0 to 24 h). Primer sequences are provided in Table S10. Reactions were performed in quintuplicate on a Roche LightCycler 480 using ChamQ SYBR qPCR Master Mix (Vazyme). Expression levels were normalized to the housekeeping gene β-actin (*ACTB*), and relative fold changes were calculated using the 2^−ΔΔCt^ method.

### Statistical analysis

All experiments were performed with at least three independent replicates. Data are presented as mean ± SD and were analyzed using GraphPad Prism software (v9.5). For comparisons among multiple groups, statistical significance was determined by one-way ANOVA, followed by Tukey's post hoc test.

## Supplementary Material

msag144_Supplementary_Data

## Data Availability

All data are available in the main text or the Supplementary materials.
