# Supplementary material for: Adaptive evolution of endothelial nitric oxide synthase (NOS3) may reduce cetacean susceptibility to decompression sickness
Source: Mol Biol Evol. 2026 Jun 11;43(6):msag144. doi: 10.1093/molbev/msag144 (PMC13308534; doi:10.1093/molbev/msag144)
Supplement: msag144_Supplementary_Data [file msag144_supplementary_data.zip › SI v1-0414.docx]

**Supplemental Information**

**Adaptive evolution of endothelial nitric oxide synthase (NOS3) may reduce cetacean susceptibility to decompression sickness**

Ran Tian^1,*^, Liang Zhao^1^, Tinghui Li^1^, Yinyin Yan^1^, Inge Seim^2,*^, Guang Yang^1,3,*^

^1^ Jiangsu Key Laboratory for the Biodiversity Conservation and Sustainable Utilization in the Middle and Lower Reaches of the Yangtze River Basin, College of Life Sciences, Nanjing Normal University, Nanjing, China.

^2^ Marine Mammal and Marine Bioacoustics Laboratory, Institute of Deep-sea Science and Engineering, Chinese Academy of Sciences, Sanya, China.

^3^ Southern Marine Science and Engineering Guangdong Laboratory (Guangzhou), Guangzhou, China.

Correspondence: tianrannjnu@163.com; seim@idsse.ac.cn; gyang@njnu.edu.cn

**Supporting Information Text**

1. **Anatomical and physiological adaptations of marine mammals to minimize the risk of decompression sickness**

Marine mammals have a range of unique characteristics that likely help minimize the buildup of intravascular and extravascular nitrogen gas bubbles. Bradycardia during the descent and bottom dive phases, together with a decreased ascent rate and an increase in heart rate, reduces the inert gas burden of whales and seals (Andrews, et al. 1997; Fahlman, et al. 2006; Tyack, et al. 2006). A compliant lung (i.e., highly flexible) and stiffened upper respiratory tract in marine mammals block gas exchange at depth, thereby limiting N_2_ uptake and gas emboli during breath-hold dives (Scholander 1940). These airway reinforcements also help maintain airway permeability (no gas trapping) during compression and provide an air storage site in a non-gas-exchange compartment when lung parenchyma collapses at depth (Fahlman, et al. 2017). Studies on cetaceans have also identified extensive vascular plexuses (predominantly venous) along the airways, extending into the terminal bronchus in deep-diving species (Leith 1989; Ninomiya, et al. 2005; Costidis and Rommel 2016). This engorged plexus is hypothesized to provide structural reinforcement of the airways during dives. Recent work further suggests that the pulmonary distribution of alveolar ventilation (_A_) and cardiac output/lung perfusion () in different regions of the lung could be a common trait to manage gas exchange in cetaceans and other marine mammals (Garcia Párraga, et al. 2018). Finally, it has been hypothesized that the large amounts of adipose tissue (i.e., blubber) in marine mammals could act as a N_2_ absorbent, thereby directly reducing bubble formation during dives (Fahlman 2009).

**References:**

Andrews RD, Jones D, Williams J, Thorson P, Oliver G, Costa D, Boeuf BL. 1997. Heart rates of northern elephant seals diving at sea and resting on the beach. Journal of Experimental Biology 200:2083-2095.

Costidis AM, Rommel SA. 2016. The extracranial venous system in the heads of beaked whales, with implications on diving physiology and pathogenesis. Journal of Morphology 277:34-64.

Fahlman A. 2009. How do marine mammals avoid DCS. The future of diving: 100 years of Haldane and beyond:129-135.

Fahlman A, Moore MJ, Garcia-Parraga D. 2017. Respiratory function and mechanics in pinnipeds and cetaceans. Journal of Experimental Biology 220:1761-1773.

Fahlman A, Olszowka A, Bostrom B, Jones DR. 2006. Deep diving mammals: dive behavior and circulatory adjustments contribute to bends avoidance. Respiratory physiology & neurobiology 153:66-77.

Garcia Párraga D, Moore M, Fahlman A. 2018. Pulmonary ventilation–perfusion mismatch: a novel hypothesis for how diving vertebrates may avoid the bends. Proceedings of the Royal Society B: Biological Sciences 285:20180482.

Leith D. 1989. Adaptations to deep breath-hold diving: respiratory and circulatory mechanics. Undersea Biomedical Research 16:345-354.

Ninomiya H, Inomata T, Shirouzu H, Katsumata E. 2005. Microanatomy of the terminal air spaces of Baird's beaked whale (Berardius bairdii) lungs. Journal of veterinary medical science 67:473-479.

Scholander PF. 1940. Experimental investigations on the respiratory function in diving mammals and birds. Hvalradets skrifter 22:1.

Tyack PL, Johnson M, Soto NA, Sturlese A, Madsen PT. 2006. Extreme diving of beaked whales. Journal of Experimental Biology 209:4238-4253.

**Supplementary Figures**


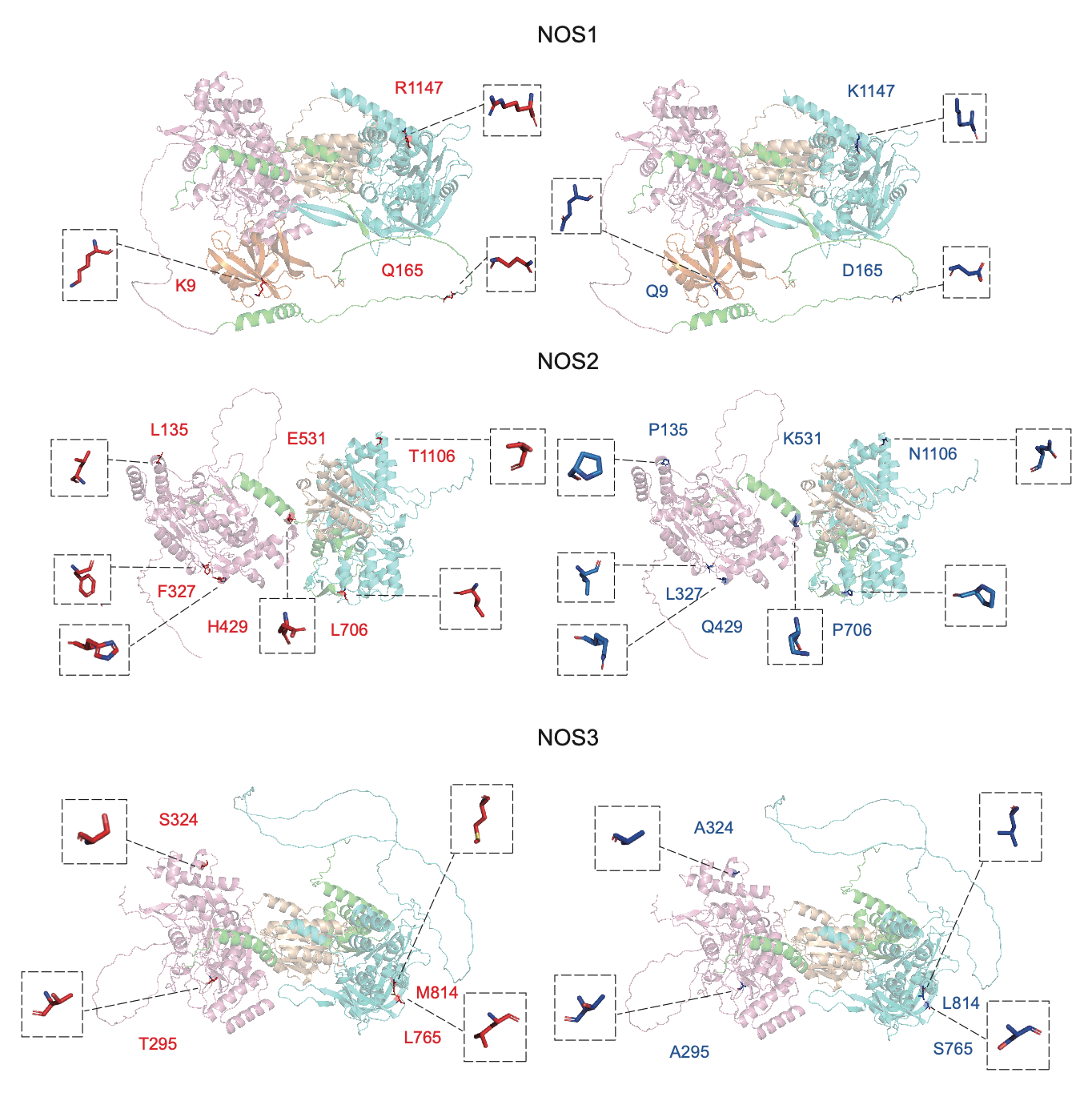


**Figure S1** AlphaFold3-predicted structures of cetacean NOS proteins. Left, native bottlenose dolphin form. Right, bottlenose dolphin sequences changed to human at cetacean-specific amino acid residues. The residues altered are highlighted in red (left) and blue (right).


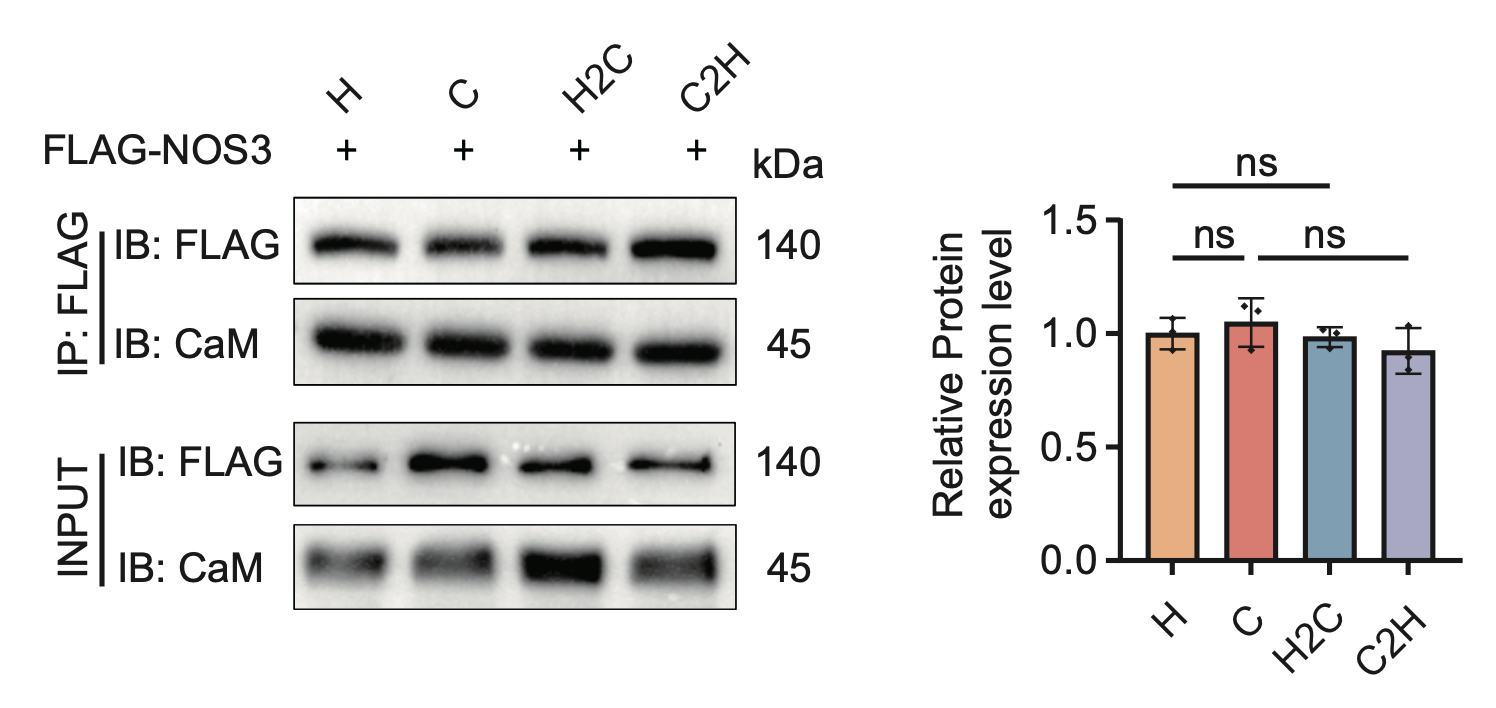


**Figure S2 Co-IP analysis of NOS3 and CaM.**

Left, co-immunoprecipitation (Co-IP) of FLAG-tagged NOS3 constructs with endogenous calmodulin (CaM) in COS7 cells. Molecular weight markers (kDa) are indicated. H denotes human *NOS3*, C, cetacean *NOS3*; the expression constructs H2C and C2H code for NOS3 proteins where the four cetacean-specific amino acids in Fig. 1D were replaced by their corresponding counterparts. Right, densitometric quantification of the co-IP results from three independent experiments (mean ± S.D.). Data points represent biological replicates. (ns denotes not significant; ^****^*P* < 0.0001, ^***^*P* < 0.001, ^**^*P* < 0.01, ^*^*P* < 0.05; full details in Table S6). Full images of blots in Supplementary Fig. S7.


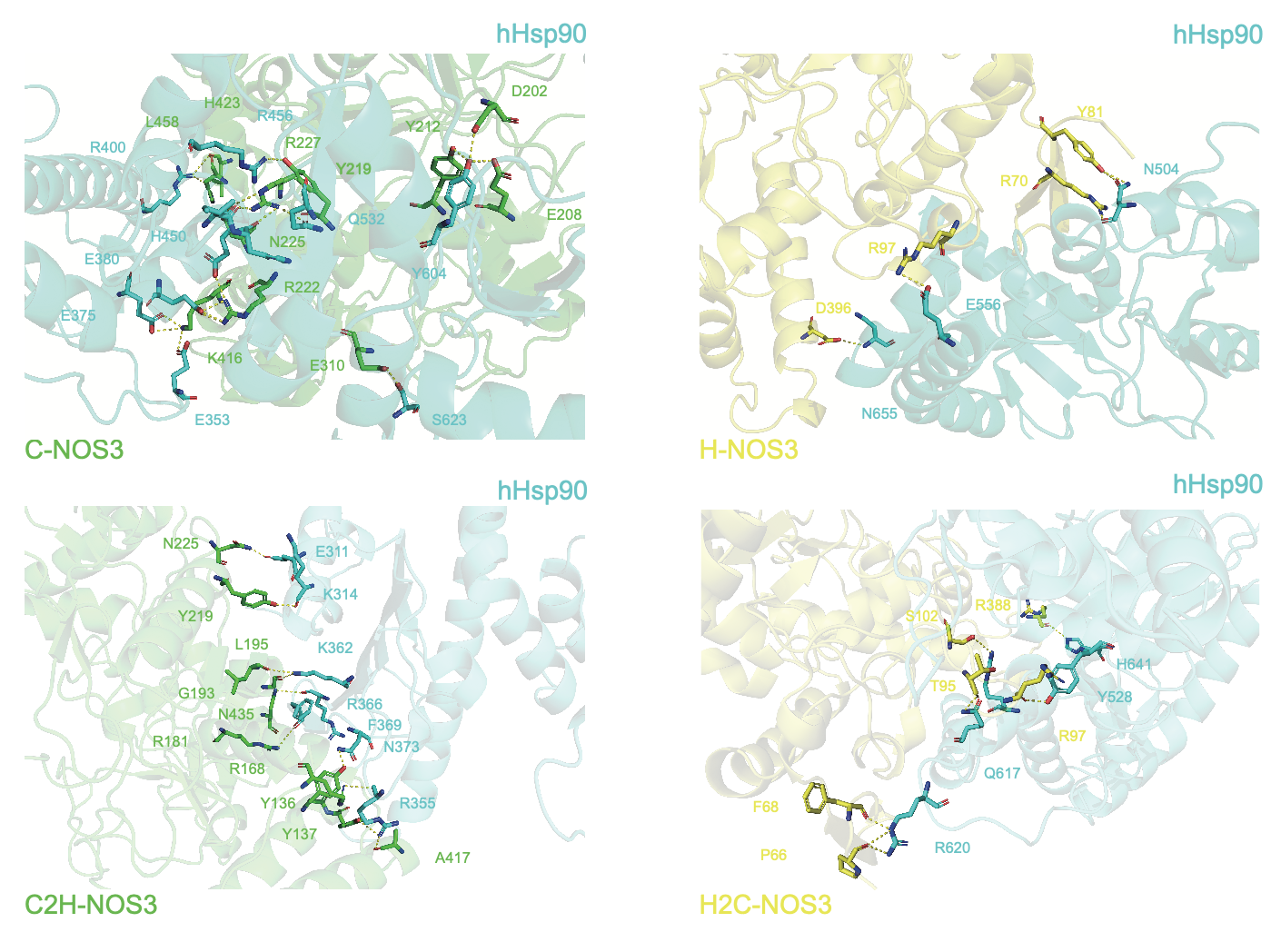


**Figure S3** **Predicted interactions between human Hsp90 (hHsp90) and eNOS (NOS3).** Shown, clockwise from the top, are: bottlenose dolphin (C-NOS3), human (H-NOS3), and reciprocal chimeras in which the four cetacean-specific residues were swapped to generate a “humanized” cetacean sequence (C2H-NOS3), and a “cetaceanized” human sequence (H2C-NOS3). Dashed lines represent hydrogen bonds. Three-dimensional protein structures were predicted with AlphaFold3. The molecular docking analyses were conducted by HDOCK, followed by refinement using RossettaDock for fine-tuning. The human Hsp90 isoform HSP90AA1 is shown.


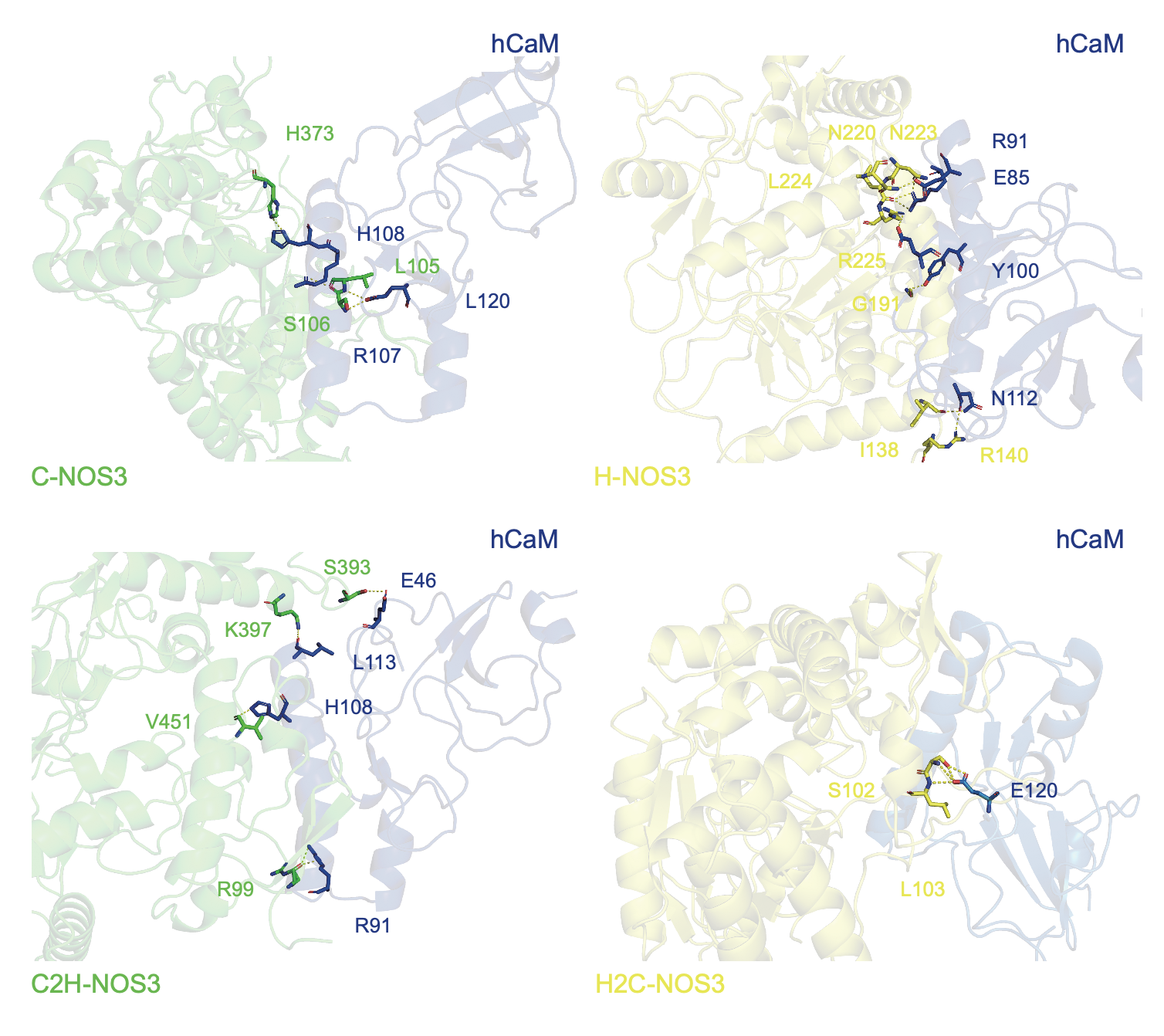


**Figure S4** **Predicted interactions between human CaM (hCaM) and eNOS (NOS3).** Shown, clockwise from the top, are: bottlenose dolphin (C-NOS3), human (H-NOS3), and reciprocal chimeras in which the four cetacean-specific residues were swapped to generate a “humanized” cetacean sequence (C2H-NOS3), and a “cetaceanized” human sequence (H2C-NOS3). Dashed lines represent hydrogen bonds. Three-dimensional protein structures were predicted with AlphaFold3. The molecular docking analyses were conducted by HDOCK, followed by refinement using RossettaDock for fine-tuning. The human CaM isoform CALM1 is shown.

**Figure S5 Expression of key apoptosis-related markers in HUVECs after 12 h of hypoxia (1% O₂) measured by qRT-PCR.**

**A)** Anti-apoptotic Bcl-2 (*BCL2*). **B)** Pro-apoptotic Bax (*BAX*). **C)** Apoptosis effector caspase 3 (*CASP3*). NC denotes empty vector control (*pcDNA3.1*), H, human *NOS3*; C, cetacean *NOS3*; the expression constructs H2C and C2H code for NOS3 proteins where the four cetacean-specific amino acids in Figure 1D were replaced by their corresponding counterparts. Data presented as mean arbitrary density units ± S.D., normalized to empty vector control at normoxia (i.e., ~21% O_2_), with individual data points overlaid (ns denotes not significant; ^****^*P* < 0.0001, ^***^*P* < 0.001, ^**^*P* < 0.01, ^*^*P* < 0.05; full details in Table S6). Expression levels were normalized to β-actin (*ACTB*).


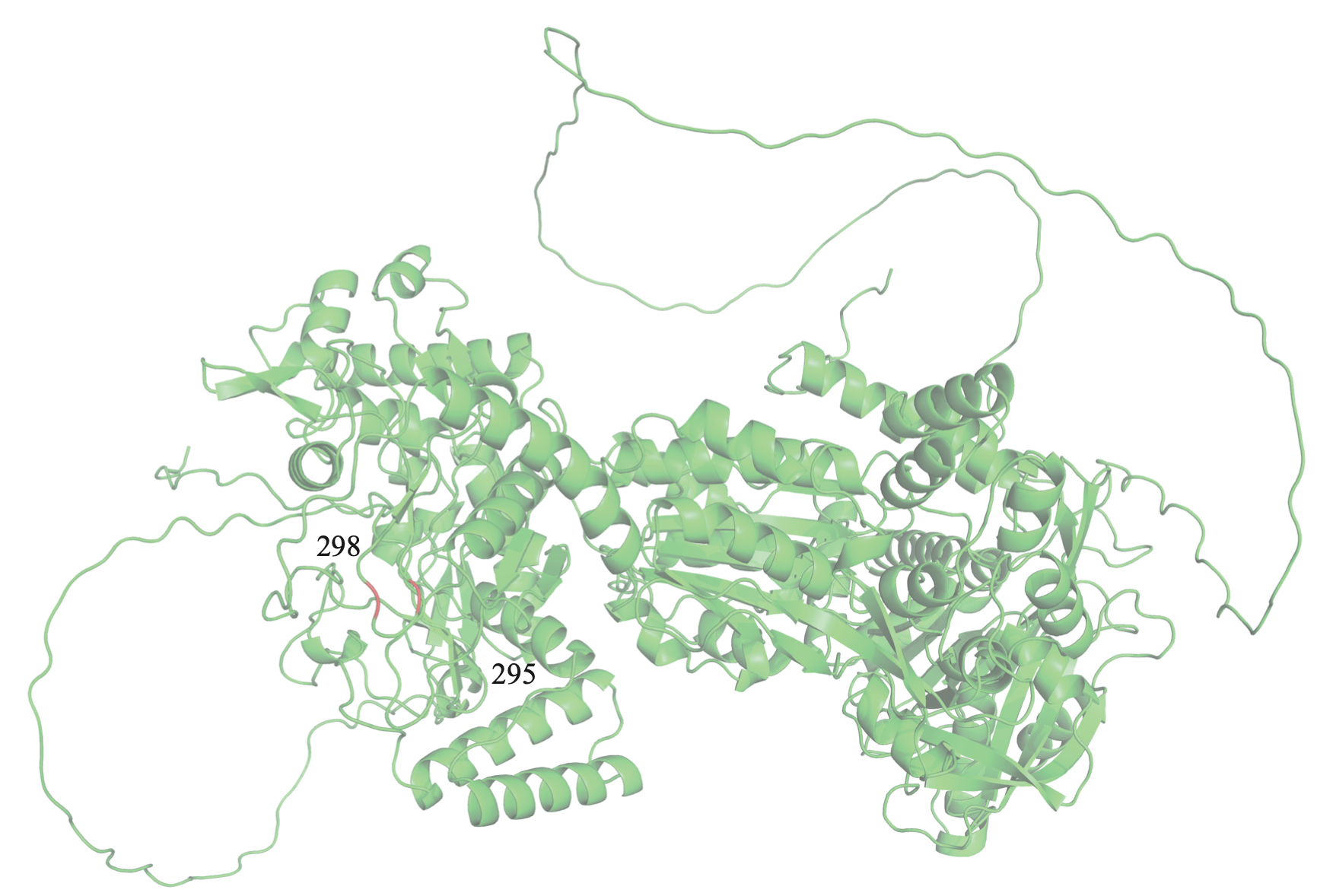


**Figure S6** AlphaFold3-predicted structures of site 295 and 298 of cetacean NOS3 proteins.

**Figure S7 Uncropped Western blot images.** The corresponding main text figures (Fig. 2B, 2C, 3A, 4A, 4C) and supplementary figure (Fig. S2) are indicated at the top left.
